# Effect of Aneurysm and Patient Characteristics on Intracranial Aneurysm Wall Thickness

**DOI:** 10.3389/fcvm.2021.775307

**Published:** 2021-12-08

**Authors:** Jason M. Acosta, Anne F. Cayron, Nicolas Dupuy, Graziano Pelli, Bernard Foglia, Julien Haemmerli, Eric Allémann, Philippe Bijlenga, Brenda R. Kwak, Sandrine Morel

**Affiliations:** ^1^Department of Pathology and Immunology, Faculty of Medicine, University of Geneva, Geneva, Switzerland; ^2^School of Pharmaceutical Sciences, University of Geneva, Geneva, Switzerland; ^3^Institute of Pharmaceutical Sciences of Western Switzerland, University of Geneva, Geneva, Switzerland; ^4^Neurosurgery Division, Department of Clinical Neurosciences, Faculty of Medicine, Geneva University Hospitals and University of Geneva, Geneva, Switzerland

**Keywords:** intracranial aneurysm, subarachnoid hemorrhage, risk factors, wall thickness uniformity, wall thickness

## Abstract

**Background:** The circle of Willis is a network of arteries allowing blood supply to the brain. Bulging of these arteries leads to formation of intracranial aneurysm (IA). Subarachnoid hemorrhage (SAH) due to IA rupture is among the leading causes of disability in the western world. The formation and rupture of IAs is a complex pathological process not completely understood. In the present study, we have precisely measured aneurysmal wall thickness and its uniformity on histological sections and investigated for associations between IA wall thickness/uniformity and commonly admitted risk factors for IA rupture.

**Methods:** Fifty-five aneurysm domes were obtained at the Geneva University Hospitals during microsurgery after clipping of the IA neck. Samples were embedded in paraffin, sectioned and stained with hematoxylin-eosin to measure IA wall thickness. The mean, minimum, and maximum wall thickness as well as thickness uniformity was measured for each IA. Clinical data related to IA characteristics (ruptured or unruptured, vascular location, maximum dome diameter, neck size, bottleneck factor, aspect and morphology), and patient characteristics [age, smoking, hypertension, sex, ethnicity, previous SAH, positive family history for IA/SAH, presence of multiple IAs and diagnosis of polycystic kidney disease (PKD)] were collected.

**Results:** We found positive correlations between maximum dome diameter or neck size and IA wall thickness and thickness uniformity. PKD patients had thinner IA walls. No associations were found between smoking, hypertension, sex, IA multiplicity, rupture status or vascular location, and IA wall thickness. No correlation was found between patient age and IA wall thickness. The group of IAs with non-uniform wall thickness contained more ruptured IAs, women and patients harboring multiple IAs. Finally, PHASES and ELAPSS scores were positively correlated with higher IA wall heterogeneity.

**Conclusion:** Among our patient and aneurysm characteristics of interest, maximum dome diameter, neck size and PKD were the three factors having the most significant impact on IA wall thickness and thickness uniformity. Moreover, wall thickness heterogeneity was more observed in ruptured IAs, in women and in patients with multiple IAs. Advanced medical imaging allowing *in vivo* measurement of IA wall thickness would certainly improve personalized management of the disease and patient care.

## Introduction

**Figure 1:**
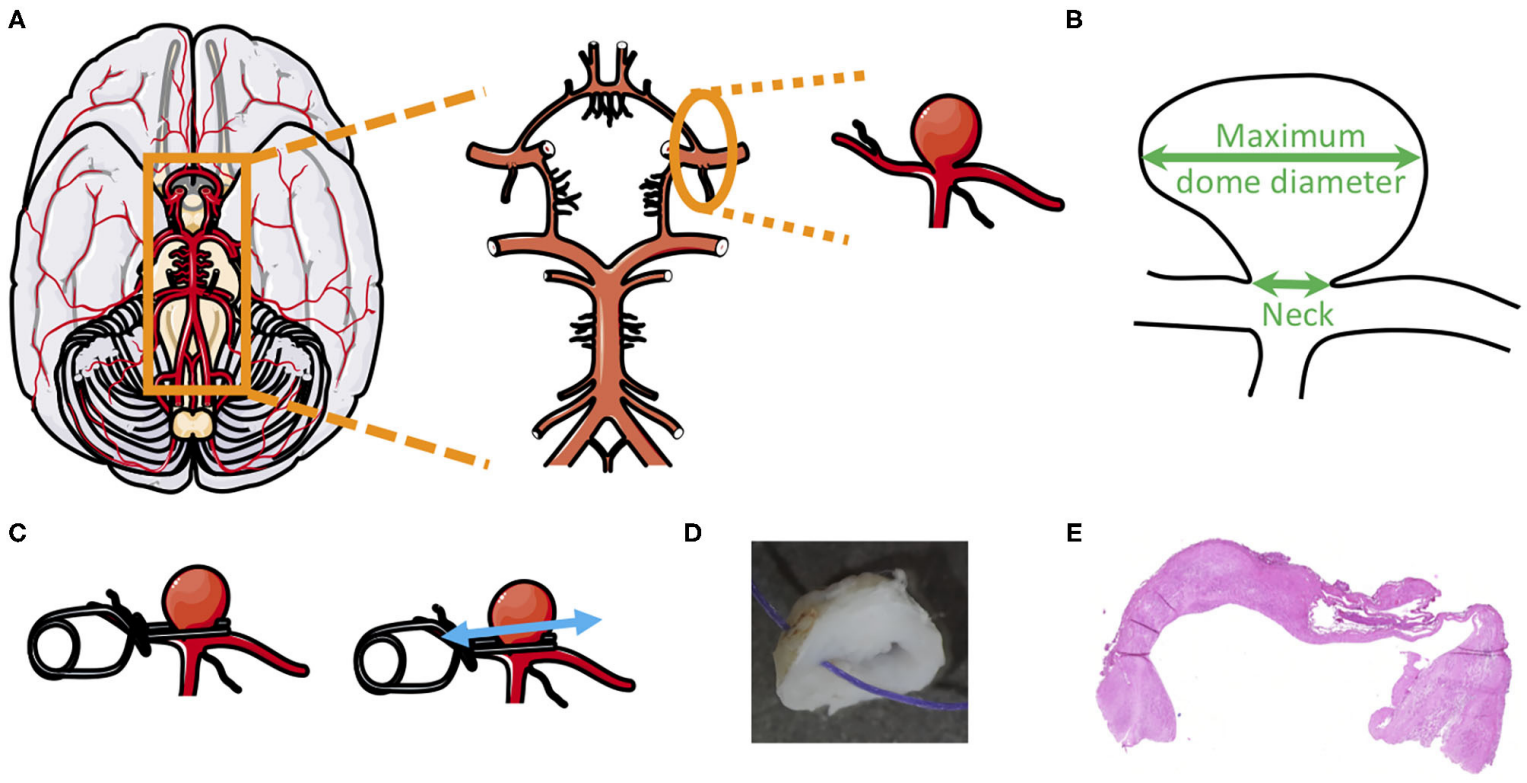
Intracranial aneurysm dome, from the circle of Willis to histology. **(A)** Example of intracranial aneurysm location in the circle of Willis. **(B)** Schematic representation of an aneurysm dome to show where neck size and maximum dome diameter were measured. **(C–E)** After clipping of the IA neck **(C)**, the IA dome was resected and fixed in formol **(D)**, cutted and stained with hematoxylin and eosin **(E)** to allow IA wall thickness measurement.

Intracranial aneurysm (IA) resulting from the local outbulging of cerebral arteries is a disease with life-threatening complications. IAs are most often observed at bifurcations of cerebral arteries in the circle of Willis, which is a network of arteries localized at the basis of the brain and allowing its perfusion (Figure 1A). IAs have usually a saccular form (Figure 1A-right panel). Once an IA has formed, it can remain stable, grow or rupture. The most severe complication of an IA is its rupture leading to subarachnoid hemorrhage (SAH) (1). In Switzerland, a recent study demonstrated that SAH is lethal in 24% of cases and causes disability in more than 50% of patients (2). In the general population, the prevalence of IAs ranges from 2 to 3% (3), with some sources indicating it might even reach 9% (4, 5). Furthermore, IA prevalence is higher in 35–60 years old patients (6), in women (7) or in patients affected by polycystic kidney disease (PKD) (8, 9). Rupture probability of IAs has been estimated between 0.3 and 15% per 5 years (1, 10) with an annual rupture rate of 1% (11). Importantly, IAs are usually asymptomatic until rupture, making this illness a silent killer. Unruptured IAs are often unexpectedly found during cranial imaging (10). The discovery of unruptured IAs results in stress and anxiety for patients who are then confronted with the difficult decision to undergo prophylactic surgery or not. A precise evaluation of rupture probability is essential to help patients and physicians in this difficult choice. However, means to accurately estimate the likelihood of rupture are currently missing. Existing prediction tools such as PHASES (12), UIAT (13), and ELAPSS (14) scores consider various risk factors commonly linked with IA rupture, such as arterial hypertension, patient age, previous SAH, co-morbidities, IA size, location and morphology. Although these scores are based on readily available clinical data and correlate well with disease severity, they have several limitations. Indeed, retrospective studies showed that these scores do not perfectly reflect the likelihood of rupture, which may lead to overtreatment of unruptured IAs (15–22). Presently, no treatment is available to prevent IA rupture and prophylactic surgery presents important risks that must be considered (10). Indeed, endovascular coiling or surgical clipping is associated with 4.8% (23) and 6.7% (24) unfavorable outcomes, respectively.

The formation and rupture of IAs is a complex pathological process that despite extensive research (25–27), is still poorly understood. Considerable scientific evidence supports the notion that hemodynamic forces acting on the vessel wall induce vascular remodeling leading to IA formation, growth and rupture (28–31). Involved processes include inflammatory cell infiltration, smooth muscle cell (SMC) phenotypic switch, apoptosis, reorganization of extra cellular matrix (ECM), calcification and lipid accumulation (28–31). Numerous studies have tackled the mysteries of IA instability based on morphological dome aspects such as IA size and intraoperative appearance (32–34). Some studies have classified aneurysmal walls as “thick” or “thin” (35, 36), but to the best of our knowledge no study has performed a quantitative analysis of IA wall thickness or a rigorous description of IA wall thickness uniformity. We believe these two factors to be of importance for IA wall instability and rupture. In this study, we have precisely measured and defined aneurysmal wall thickness and thickness uniformity on histological sections. Potential links between such IA characteristics and patient and aneurysm descriptors commonly used in clinics to determine the rupture risk of IAs were investigated.

## Materials and Methods

### Clinical Data

Patients were recruited at the Geneva University Hospitals following specific criteria. Inclusion criteria were as follows: (1) IA identified based on angiographic imaging [3D Magnetic Resonance Angiogram (3D-MRA), 3D Computed Tomography Angiogram (3D-CTA) or 3D Digital Subtraction Angiography (3D-DSA)]; (2) 18 years of age or older; and (3) patient having provided informed consent. Exclusion criteria were as follows: (1) lack of angiographic evidence for IA on 3D-MRA, 3D-CTA or 3D-DSA; (2) insufficient access to clinical data; (3) younger than 18 years of age; and 4) non-provision of informed consent. The study was approved by the Ethical Committee of the Geneva University Hospitals and by Swissethics (@neurIST protocol, ethics authorization PB_2018-00073, previously CER 07-056). All procedures were in accordance with the World Medical Association's Declaration of Helsinki.

Clinical data of recruited patients were collected with respect to IA and patient characteristics. IA characteristics were rupture status (ruptured or unruptured), vascular location, maximum dome diameter (Figure 1B), neck size (Figure 1B), and aspect (roughness, smoothness, presence of blebs and/or lobules [defined as (i) lobules have a diameter close to the IA diameter and (ii) blebs have a diameter much smaller than the IA diameter)], as previously described (27, 37, 38). Moreover, we calculated the bottleneck factor (ratio between maximum dome diameter and neck size), which is considered to be a potential predictor of IA rupture (39). Patient characteristics were age at discovery/rupture of IA, smoking status (defined as (i) never smoked more than 300 cigarettes and (ii) former (smoked more than 300 cigarettes and stopped at least 6 months ago) or current (smoked more than 300 cigarettes and continues smoking) smoker), hypertension (defined as arterial blood pressure >140/90 mmHg, regardless of treatment status), sex, ethnicity, positive family history for IA or SAH, earlier SAH, presence of multiple IAs and diagnosis of PKD.

The PHASES (12) and ELAPSS (14) scores, used to evaluate IA rupture risk and growth respectively, were calculated for all patients.

### Human Saccular IA Samples

Saccular IA samples were provided by the Division of Neurosurgery of the Geneva University Hospitals. All samples were obtained during microsurgery by resection of the IA dome (i.e., the bulging region of the IA) after clipping of the neck (Figures 1C,D). IAs were stored as previously described [Aneux Biobank (27)], fixed in formol, embedded in paraffin, sectioned at 5 μm and conserved at 4°C.

### Aneurysm Wall Thickness Measurement

**Figure 2:**
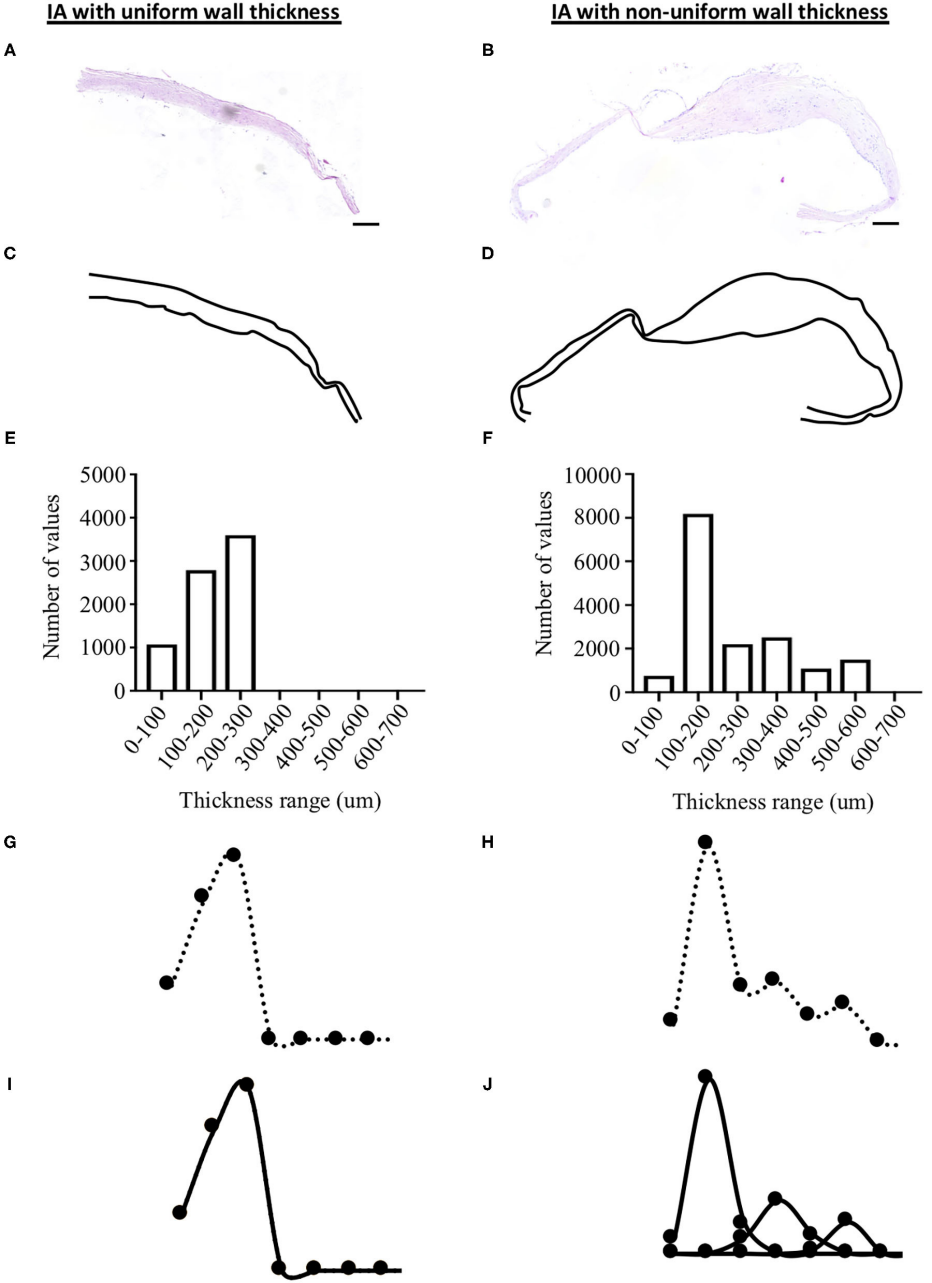
Methods for the measurement of aneurysm wall thickness and thickness uniformity. IA wall thickness was measured on hematoxylin and eosin sections **(A,B)** after drawing the external border of the IA wall **(C,D)**. A thickness topographic map of each dome was obtained **(E,F)** allowing the calculation of a Gaussian curve from this frequency distribution **(G,H)**. Each Gaussian curve was decomposed **(I,J)** to classify IA wall as uniform (i.e., 1 Gaussian curve, left side of the figure) or non-uniform (i.e., 2–5 Gaussian curves, right side of the figure). Scale bar in **(A,B)** = 200 μm.

To measure aneurysm wall thickness, aneurysmal dome sections were stained with hematoxylin and eosin (Figures 1E, 2A,B). Sections were scanned at 10× magnification in high resolution using the fully automated slide scanner Axio Scan.Z1 (Carl Zeiss Microscopy, Oberkochen, Germany). Using the software MATLAB 2019a (Mathworks, Massachusetts, USA), IA wall borders were precisely drawn (Figures 2C,D) and IA wall thickness was calculated every 0.4 μm all along the length of the resected aneurysm dome. For each sample, minimum and maximum IA wall thicknesses were extracted from all the data and the mean IA wall thickness was calculated.

### Aneurysm Wall Thickness Uniformity Measurement

Based on the thickness measurements, a thickness topographic map of each dome was obtained. Thickness values were divided into 100 μm classes from 0 to 2,000 μm and frequencies of each thickness classes were determined (Figures 2E,F). Gaussian curves from this frequency distribution were obtained for each aneurysm (Figures 2G,H). Using the Gaussian Mixture Model (GMM) in the Excel software, each aneurysm Gaussian curve was decomposed into a maximum of five simple Gaussian functions, varying according to the mean thickness, amplitude and standard deviation (Figures 2I,J). To make the best possible match between the number of Gaussian and the GMM, the Chi-2 minimization simplex method was used. Based on the number of Gaussian curves, IA walls were classified as uniform (i.e., 1 Gaussian curve, example given in Figure 2- left side) or non-uniform (i.e., 2–5 Gaussian curves, example given in Figure 2- right side).

### Statistical Analysis

Results are shown as individual values and as median ± interquartile range (IQR), as percentage or in correlations. Comparisons of medians were performed using a non-parametric Mann-Whitney *U*-test for two groups comparison and using Kruskal-Wallis and Dunn's multiple comparison tests for 4 groups comparison. Comparisons of percentages was performed using a Fisher exact test. For continuous variables with normal distribution, verified by the Kolmogonov-Smirnov test, Pearson correlations were performed to examine association between variables. For ordinal variables, Spearman correlations were performed to examine association between variables. Differences were considered statistically significant at values of *p* < 0.05.

## Results

### Characteristics of the Studied Population

The studied population was composed of 55 patients with a mean age of 54 ± 11 years. Fifty-four patients were of Eurasian ethnicity and one was African. Our study group had a majority of females (75%) and smokers (63%: 47% current smokers and 16% former smokers). Forty-two percent of patients (*N* = 23) were diagnosed with hypertension, regardless of treatment status. Positive family history of IA or SAH was declared in 18% of the patients (*N* = 10). Previous SAH was present in 9% (*N* = 5) of the patients. Multiple-aneurysm cases concerned 49% of patients (*N* = 27). Six patients (11%) were affected by PKD. Eighteen IAs (33%) were ruptured and 37 (67%) were unruptured. The mean maximum dome diameter was 6.7 ± 3.6 mm. The mean aneurysm neck size was 3.9 ± 2.4 mm. The mean bottleneck factor was 1.7 ± 0.6. The majority of the aneurysms resected for this study were located at the middle cerebral artery (MCA, *N* = 38, 69%). The other vascular locations were internal carotid artery (*N* = 1), A2 artery (*N* = 2), posterior communicating artery (*N* = 2), anterior communicating artery (*N* = 5) and anterior cerebral artery (*N* = 7). Concerning aneurysm aspects, the ratio of rough/smooth domes was 0.3 rough and 0.7 smooth; 44% of domes included blebs, 27% included lobules and 55% of them included blebs and/or lobules.

### Effects of IA Characteristics on IA Wall Thickness

**Figure 3:**
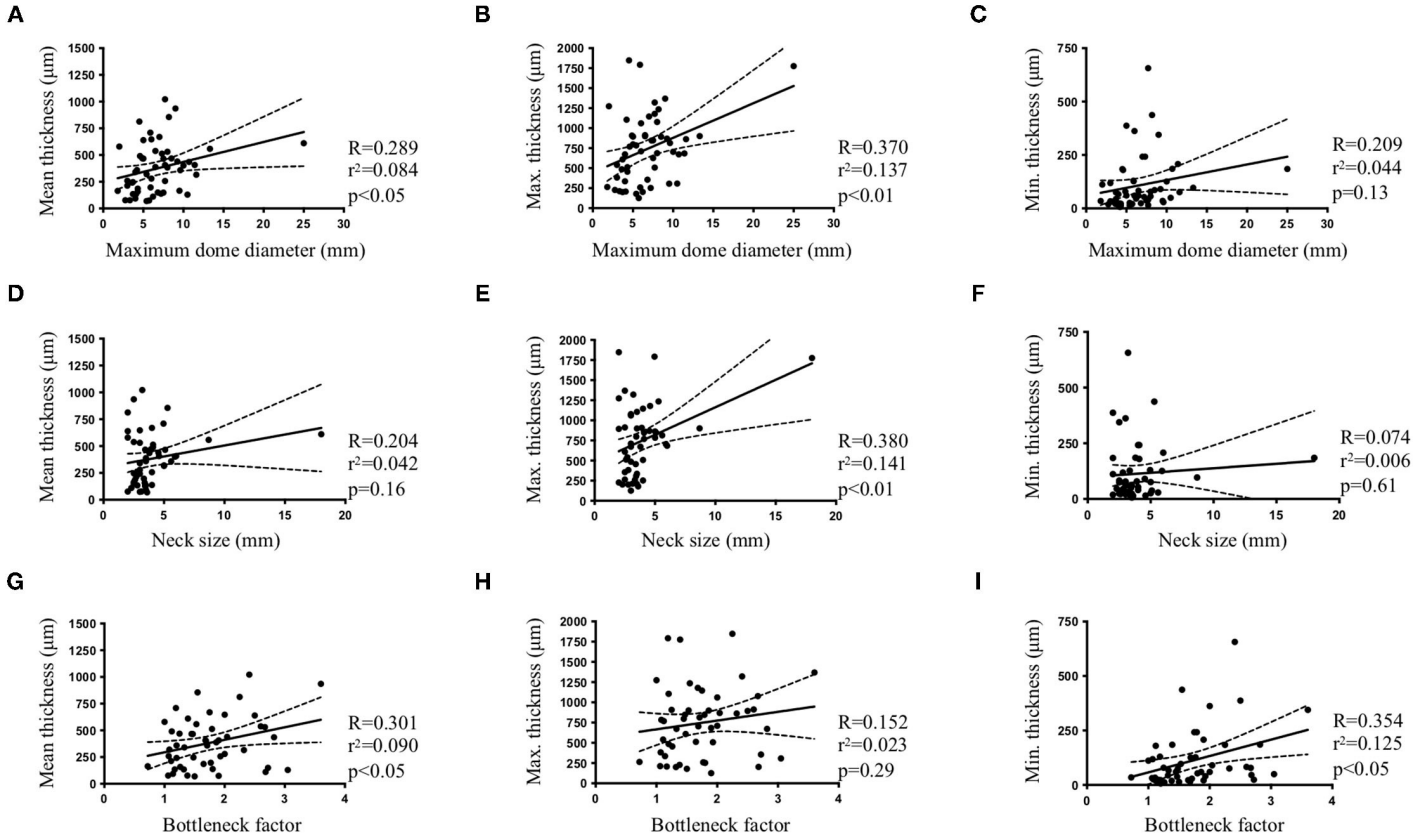
Intracranial aneurysm characteristics and IA wall thickness. Pearson correlations between maximum dome diameter **(A–C)**, neck size **(D–F)** or bottleneck factor **(G–I)** and mean **(A,D,G)**, maximum [Max. **(B,E,H)**] or minimum [Min. **(C,F,I)**] IA wall thickness.

**Figure 4:**
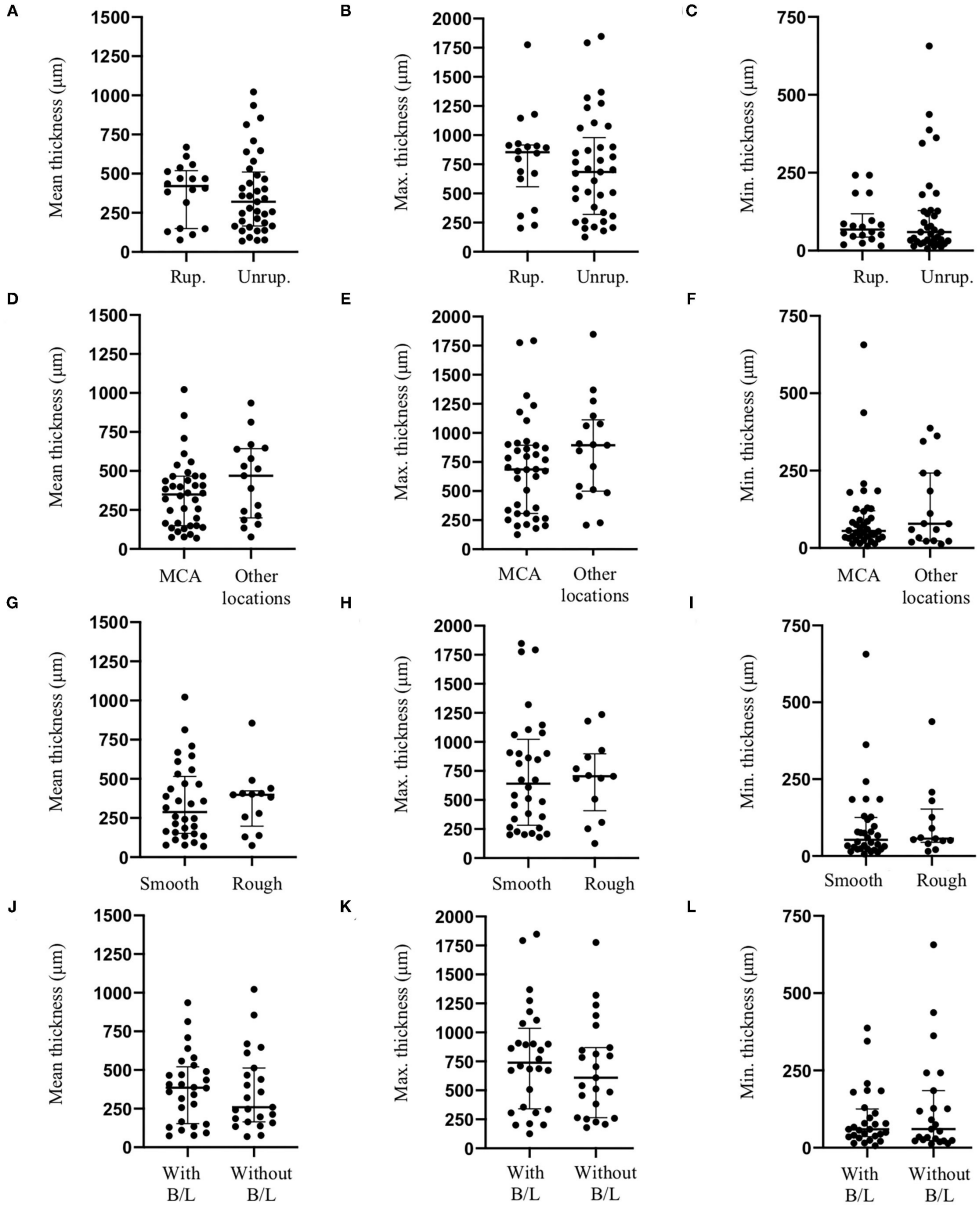
Effects of intracranial aneurysm rupture status, location, aspect and morphology on IA wall thickness. Intracranial aneurysm rupture status [Rup.: Ruptured; Unrup.: Unruptured, **(A–C)**], location **(D–F)**, aneurysm aspect **(G–I)** or morphology [presence of blebs and/or lobules (B/L), **(J–L)**] did not affect mean **(A,D,G,J)**, maximum [Max., **(B,E,H,K)**] or minimum [Min., **(C,F,I,L)**] IA wall thickness.

Maximum dome diameter was positively correlated to mean (Figure 3A) and maximum (Figure 3B) IA wall thickness. A positive correlation was also observed between neck size and maximum IA wall thickness (Figure 3E). Bottleneck factor was positively correlated to mean (Figure 3G) and minimum (Figure 3I) IA wall thickness. No correlations were observed between maximum dome diameter and minimum IA wall thickness (Figure 3C), or between neck size and mean or minimum IA wall thickness (Figures 3D,F, respectively). Furthermore, no correlation was found between bottleneck factor and maximum IA wall thickness (Figure 3H). Finally, no associations were found between IA wall thickness (mean, maximum and minimum) and IA rupture status (Figures 4A–C), IA location (Figures 4D–F), IA aspect (Figures 4G–I) or morphology (Figures 4J–L).

### Effects of IA Characteristics on IA Wall Thickness Uniformity

**Figure 5:**
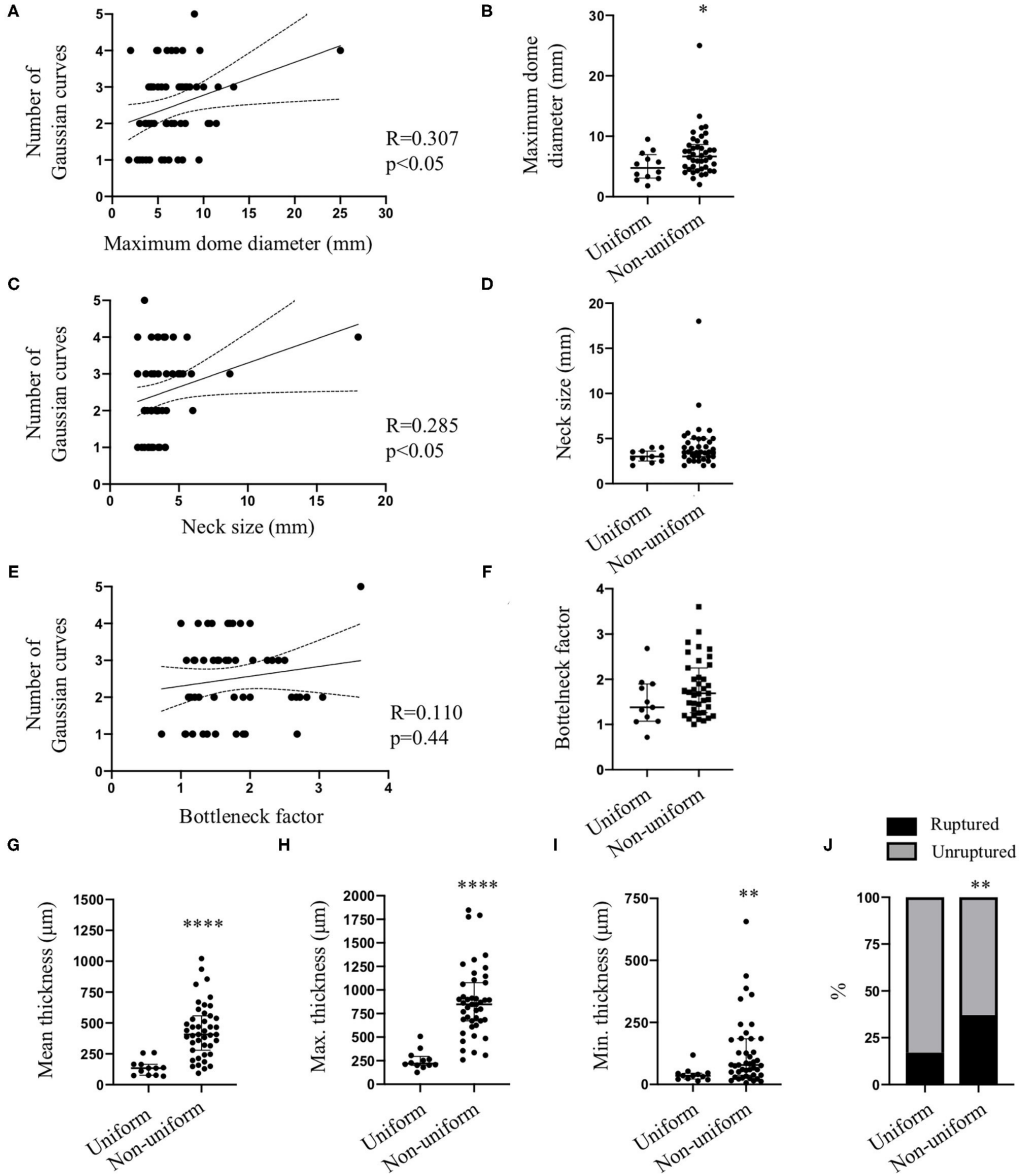
Intracranial aneurysm characteristics and wall uniformity. Spearman correlations between maximum dome diameter **(A)**, neck size **(C)** or bottleneck factor **(E)** and number of Gaussian curves characterizing each intracranial aneurysm. Maximum dome diameter **(B)**, neck size **(D)**, and bottleneck factor **(F)** values in groups having a uniform or non-uniform IA wall thickness. Mean **(G)**, maximum [Max. **(H)**] and minimum [Min. **(I)**] wall thickness in groups having a uniform or non-uniform IA wall thickness. Data are shown as individual values and as median with interquartile range. *p < 0.05, **p < 0.01, ****p < 0.0001, non-parametric Mann-Whitney *U*-test. **(J)** Distribution of ruptured (black) and unruptured (gray) IA domes between uniform and non-uniform wall thickness groups. **p < 0.01, Fisher's exact test.

IA wall thickness uniformity was based on the number of Gaussian curves characterizing each aneurysm wall. For twelve IA domes, one Gaussian curve (example given in Figure 2I) characterized the aneurysm wall meaning that these walls had a uniform thickness. For forty-three IA domes, the IA wall thickness was depicted by 2–5 Gaussian curves (example given in Figure 2J) implying that they had different degrees of non-uniform wall thickness. Maximum dome diameter (Figure 5A) and neck size (Figure 5C) were positively correlated with the number of Gaussian curves characterizing each IA wall. No correlation was found between the bottleneck factor and the number of Gaussian curves (Figure 5E). IA walls classified as uniform showed a lower maximum dome diameter in comparison with IA walls classified as non-uniform (Figure 5B). No difference with respect to neck size (Figure 5D) or bottleneck factor (Figure 5F) was shown between uniform and non-uniform IA walls. Mean (Figure 5G), maximum (Figure 5H) and minimum (Figure 5I) IA wall thickness were lower in uniform walls in comparison with walls with a non-uniform thickness. The proportion of unruptured IAs was higher in the IA group with uniform wall thickness (*N* = 10/12) in comparison with the IA group with non-uniform wall thickness (*N* = 27/43) (Figure 5J). This outcome was not induced by differences in maximum dome diameter, neck size, or bottleneck factor between unruptured IA domes with uniform wall thickness and those with non-uniform wall thickness (Table 1). In the group of IAs with uniform wall thickness, maximum dome diameter was not different between unruptured and ruptured IAs (Table 1). In the group of IAs with non-uniform wall thickness, maximum dome diameter was significantly higher in ruptured IAs in comparison with unruptured IAs (*p* < 0.05, Table 1). No differences for neck size and bottleneck factor were found between ruptured and unruptured IAs (Table 1). The distribution of IA domes with smooth/rough aspect or with presence or absence of blebs and/or lobules were not different between IA domes having a uniform or a non-uniform wall thickness (data not shown). In the group of IA domes with a uniform IA wall thickness, the proportion of IAs located on the MCA was higher than in the group of IA domes with a non-uniform wall thickness (83 *vs*. 65%, *p* < 0.01). Maximum dome diameter and bottleneck factor were not different between IA domes located in MCA or located at other locations, and were not different between IA domes with a uniform or non-uniform wall thickness (Table 1). In the group of IAs with uniform wall thickness, neck size was not different between MCA-located IA domes and non-MCA-located IA domes (Table 1). However, in the non-uniform wall thickness group, neck size was significantly higher (*p* < 0.01) in MCA-located IA domes in comparison with non-MCA-located IA domes (Table 1).

**Table 1:** Maximum (Max.) dome diameter, neck size and bottleneck factor according to IA rupture status, IA location, IA multiplicity, patient sex, and PKD diagnosis in groups with uniform or non-uniform wall thickness.

			**Uniform wall thickness**	**Non-uniform wall thickness**
IA rupture status	Unruptured IA	Max. dome diameter	4.8 (3.2–7.3)	5.9 (4.2–8.0)
		Neck size	3.1 (2.8–3.8)	3.3 (2.7–5.0)
		Bottleneck factor	1.3 (1.1–1.8)	1.7 (1.2–2.0)
	Ruptured IA	Max. dome diameter	4.5 (2.8–6.2)	7.7 (6.8–10.7)
		Neck size	2.2 (2.0–2.3)	3.9 (3.4–4.9)
		Bottleneck factor	2.0 (1.4–2.7)	1.8 (1.5–2.7)
IA location	MCA	Max. dome diameter	5.6 (3.2–7.3)	7.7 (5.4–10.0)
		Neck size	3.0 (2.6–3.8)	4.3 (3.7–5.2)
		Bottleneck factor	1.5 (1.1–1.9)	1.6 (1.3–2.2)
	Other locations	Max. dome diameter	3.4 (2.8–4.1)	5.0 (4.3–7.0)
		Neck size	2.7 (2.0–3.5)	3.0 (2.5–3.5)
		Bottleneck factor	1.3 (1.2–1.4)	1.8 (1.2–2.2)
IA multiplicity	Unique IA	Max. dome diameter	5.4 (3.4–7.5)	7.7 (6.0–9.8)
		Neck size	3.2 (2.4–3.9)	3.5 (3.0–5.0)
		Bottleneck factor	1.4 (1.2–1.9)	1.9 (1.2–2.7)
	Multiple IAs	Max. dome diameter	3.3 (1.8–5.7)	5.7 (4.4–8.0)
		Neck size	3.0 (2.5–3.1)	3.5 (2.6–4.5)
		Bottleneck factor	1.1 (0.7–1.9)	1.7 (1.3–1.9)
Sex	Women	Max. dome diameter	5.7 (3.3–7.2)	7.2 (5.0–8.6)
		Neck size	3.1 (2.5–4.0)	3.5 (3.0–5.0)
		Bottleneck factor	1.8 (1.1–1.9)	1.8 (1.5–2.4)
	Men	Max. dome diameter	3.7 (2.9–6.8)	4.4 (4.9–8.4)
		Neck size	2.8 (2.2–3.3)	3.5 (2.8–4.9)
		Bottleneck factor	1.2 (1.1–1.4)	1.3 (1.2–1.9)
PKD diagnosis	PKD	Max. dome diameter	3.6 (2.1–6.4)	3.9 (3.7–4.0)
		Neck size	3.2 (2.6–3.9)	3.0 (2.5–3.5)
		Bottleneck factor	1.1 (0.9–1.6)	1.3 (1.1–1.5)
	Non-PKD	Max. dome diameter	5.6 (3.4–7.3)	6.9 (4.9–8.9)
		Neck size	3.0 (2.3–3.6)	3.5 (3.0–5.0)
		Bottleneck factor	1.5 (1.3–1.9)	1.7 (1.3–2.3)

*Values are median (Interquartile range)*.

### Effects of Patient Characteristics on IA Wall Thickness and Thickness Uniformity

**Figure 6:**
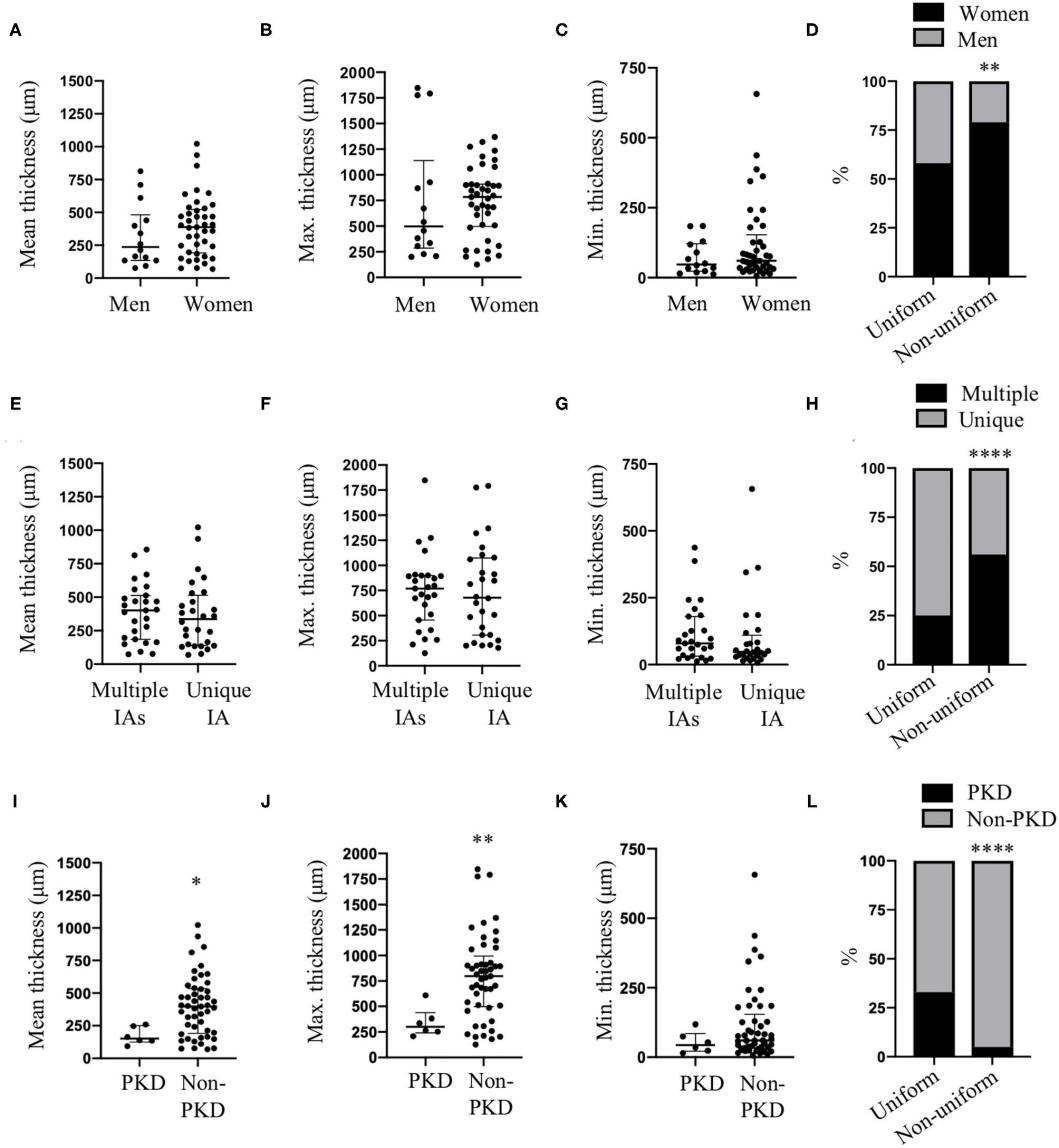
Sex, IA multiplicity and PKD effects on IA wall thickness and uniformity. Mean **(A,E,I)**, maximum [Max. **(B,F,J)**] and minimum [Min. **(C,G,K)**] IA wall thickness in men and women **(A–C)**, for groups of multiple IAs and unique IA **(E–G)** and in patients diagnosed or not with PKD **(I–K)**. Data are shown as individual values and as median with interquartile range. *p < 0.05, **p < 0.01, non-parametric Mann-Whitney *U*-test. **(D)** Distribution of women (black) and men (gray) between uniform and non-uniform wall thickness groups. **(H)** Distribution of multiple IAs (black) and unique IA (gray) between uniform and non-uniform wall thickness groups. **(L)** Distribution of PKD (black) and non-PKD (gray) patients between uniform and non-uniform wall thickness groups. **p < 0.01, ****p < 0.0001, Fisher's exact test.

None of the patient characteristics (age, sex, hypertension, smoking status, positive family history, or presence of multiple IAs) had an effect on mean, maximum or minimum IA wall thickness (data not shown). Although, no differences for mean (Figure 6A), maximum (Figure 6B) or minimum (Figure 6C) wall thickness were observed between men and women, the proportion of women was higher in the IA group with non-uniform wall thickness (*N* = 34/43, 79%) in comparison with the group of IAs with uniform wall thickness (*N* = 7/12, 58%) (Figure 6D). This observation was not induced by differences in maximum dome diameter, neck size or bottleneck factor between women's IA domes with uniform wall thickness and those with non-uniform wall thickness (Table 1). Also, no significant differences were found for maximum dome diameter, neck size or bottleneck factor between men's IA domes in the uniform group and those in the non-uniform group (Table 1). Furthermore, no difference was found for maximum dome diameter, neck size or bottleneck factor when comparing men and women in uniform and non-uniform wall thickness groups (Table 1). Existence of multiple IAs did not affect the mean (Figure 6E), maximum (Figure 6F) or minimum (Figure 6G) IA wall thickness, but their proportion was higher in the group of IAs with non-uniform wall thickness (*N* = 24/43, 56%) in comparison with the group of IAs with uniform wall thickness (*N* = 3/12, 25%) (Figure 6H). No difference was found for maximum dome diameter, neck size or bottleneck factor when comparing patients with unique or multiple IAs in uniform and non-uniform wall thickness groups (Table 1). Mean (Figure 6I) and maximum (Figure 6J) wall thickness were significantly lower in IA walls of patients affected by PKD in comparison with non-PKD patients. No difference was found for minimum wall thickness between PKD and non-PKD patients (Figure 6K). The proportion of PKD patients was higher in the group of IAs with uniform wall thickness (*N* = 4/12, 33%) in comparison with the group of IAs with non-uniform wall thickness (*N* = 2/43, 5%) (Figure 6L). Although there was a tendency to a lower maximum dome diameter of the IAs of PKD patients, no significant differences were found for maximum dome diameter, neck size or bottleneck factor between PKD and non-PKD patients taking into account the uniformity or non-uniformity of the IA wall (Table 1). The age of the patients was not different between the uniform (48 (45–54) years old) and non-uniform (55 (45–65) years old) groups. The proportion of IA domes between uniform and non-uniform groups were not affected by smoking status (smokers: uniform = 67%, non-uniform = 63%), hypertension (yes: uniform = 42%, non-uniform = 42%) or positive family history (yes: uniform = 25%, non-uniform = 16%).

### Clinical Prognosis Scores and IA Wall Thickness Uniformity

**Figure 7:**
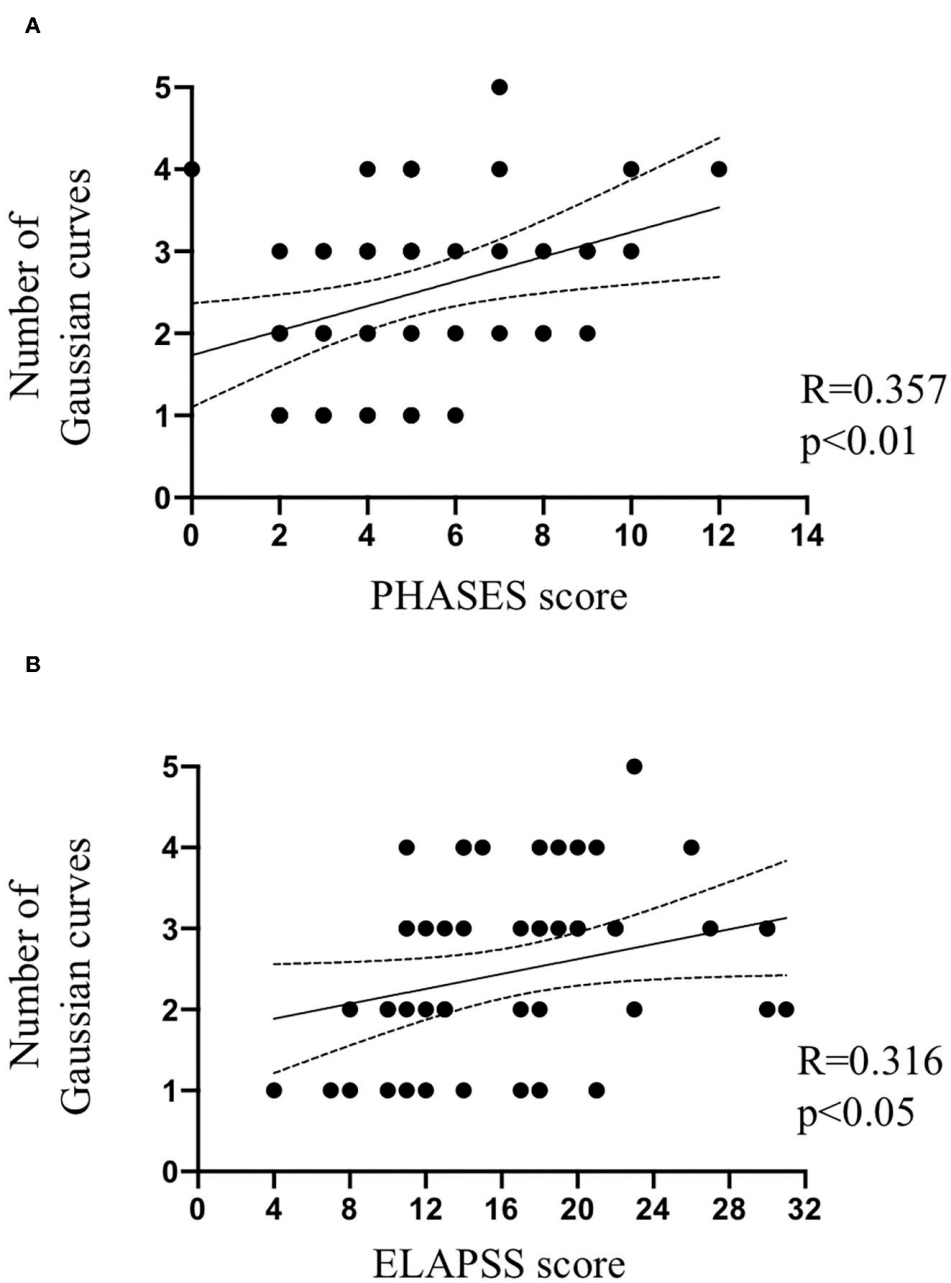
IA wall uniformity and clinical scores. Spearman correlations between PHASES score **(A)** or ELAPSS score **(B)** and the number of Gaussian curves characterizing IA wall uniformity.

The PHASES score (12), based on population, hypertension status, age, size of the aneurysm, earlier SAH from another IA and site of aneurysm was calculated for 54 patients; IA size was missing for one patient resulting in omission of this patient for this comparison. The ELAPSS score (14), based on earlier SAH, location of the IA, age, population ethnicity, size and shape of the IA was calculated for 51 patients; IA size was missing for one patient and presence of irregularities was not described for 3 patients. Interestingly, the PHASES (Figure 7A) and ELAPSS (Figure 7B) scores were positively correlated with the number of Gaussian curves characterizing IA wall uniformity.

## Discussion

Formation, growth, remodeling, destabilization and rupture of IAs are complex pathological processes. Prediction tools such as PHASES (12) and ELAPSS (14) scores suggest age, smoking status, hypertension, or aneurysm location to be strong predictors of rupture risk. In 2017, a systematic review performed by Kleinloog et al. (40) including 102 studies and describing 144 risk factors for IA rupture found strong evidence that changes in some morphological factors, such as aspect ratio, size ratio or bottleneck factor, increase IA rupture risk. Previous studies on IA wall histological features focused on the effects of patients and aneurysms characteristics on wall cellular content and ECM protein organization (26, 27, 31, 41), but few investigated IA wall thickness or thickness uniformity (35, 36). As SAH is induced by a breach in the vessel wall, precise analyses of IA wall thickness and wall thickness uniformity are paramount in grasping what makes IAs prone to rupture. Through wall thickness assessment of IA domes from the Aneux biobank, we demonstrate in this study that maximum dome diameter, neck size and diagnosis of PKD are the main factors correlated with IA wall thickness and IA wall thickness uniformity. Interestingly, IAs with a non-uniform wall thickness are more often observed in the ruptured group, in women and in patients harboring multiple IAs. Finally, PHASES and ELAPSS scores are positively correlated with IA wall thickness heterogeneity.

One of our crucial findings is that IA wall thickness and thickness heterogeneity, measured in detail on histological sections, increase with maximum dome diameter, neck size or diagnosis of PKD. These results further support a previous study performed by Kadasi et al. (35) showing morphologically that large aneurysms (>7 mm) contained a larger proportion of thick wall than thin translucent wall. This size-pathogenesis interconnection was first put forward by Asari and Ohmoto (32) who characterized a group of entirely thick-walled IAs, all having a diameter over 9 mm. Aneurysm and neck size are important parameters modulating the impact of hemodynamic forces on the IA wall (30, 42, 43). Indeed, altered cyclic circumferential stretch was associated with reduced SMC viability and collagen expression (44). Otherwise, pathological levels and patterns of wall shear stress (WSS) have been linked to endothelial cell dysfunction, phenotypic changes in SMCs, remodeling of ECM, and activation of inflammatory pathways (42, 45). In a cohort of patients with small (<10 mm) or large/giant (>10 mm) aneurysms, Schnell et al. (43) demonstrated in 2014 that larger IAs were subjected to higher WSS than smaller IAs. More recently, Cebral et al. (46) showed that high average WSS and pressure were more likely associated with thin IA wall regions, and that hyperplastic regions had lower average WSS and pressure than normal regions. Important cellular sensors of WSS are primary cilia. Patients carrying a mutation of genes affecting the expression or function of primary cilia are more prone to develop IA than the general population (8, 9). In a previous study (47), we showed that the wall of unruptured IAs from PKD patients contained less collagen than the ones of non-PKD patients. In addition, PKD IAs displayed a more degraded vascular wall phenotype comparable to what was observed in ruptured IAs. Interestingly, we also showed that the expression of the junction protein Zonula Occludens-1 (ZO-1) was reduced in endothelial cells of PKD patients in comparison with non-PKD patients. In subsequent *in vitro* experiments, we showed that the decreased expression of ZO-1 led to increased endothelial cell permeability suggesting that disturbed expression of ZO-1 in human IAs could underly the leakiness of the endothelium observed in PKD patients. Modification of IA wall composition in PKD patients may participate in the thinning of the aneurysmal wall observed in the present study. Altogether, these studies suggest that the association between IA wall thickness and morphological parameters may depend on local hemodynamic forces.

Whereas, smoking status, hypertension, aneurysm aspect, morphology, or location are risk factors used in current rupture prediction tools (12–14), we did not find any association between these parameters and IA wall thickness or thickness uniformity. Cigarette smoke is known to induce endothelial dysfunction, SMC phenotypic modulation or death, and promotes inflammation (48), which could all increase the risk of IA rupture. We have previously shown that the IA walls of smokers contained less SMCs than the ones of non-smokers and that this lower SMC content is similar to the one measured in ruptured IAs (27), strongly suggesting that reduced SMC content in the IA wall is associated with a higher risk of rupture (41). In the present study, IA wall thickness and thickness uniformity is not different between smokers and non-smokers indicating that although a lower presence of SMCs favors rupture, it does not necessarily lead to a thinner or non-uniform IA wall. Cardiovascular remodeling via SMCs migration, proliferation or hypertrophy has been shown to involve the renin-angiotensin system (49, 50). Ohkuma et al. (51), proposed that increased hemodynamic stress may activate local renin-angiotensin system resulting in arterial wall thickening, and demonstrated that the expression of angiotensin-converting enzyme, angiotensin type 1 receptor and angiotensin II were reduced in IA walls in comparison to control arteries. However, no difference in the expression of such proteins was found between patients with or without hypertension, suggesting that the local renin-angiotensin system is not activated in the case of IAs. This may explain why in our cohort no difference has been found between normotensive and hypertensive patients for IA wall thickness and thickness uniformity. Even if morphological observation of irregularities and presence of blebs and/or lobules were expected to have an impact on IA wall thickness and thickness uniformity in histological sections, no associations were found in our study. One important limitation concerning the analysis of the effects of these IA characteristics on wall thickness is that, due to IA neck clipping, we do not have access to the complete IA for histological investigations which can slightly skew the analysis. IA location is a central factor for aneurysm rupture risk (12, 13, 52). Here, IAs located in the MCA seemed to possess a more uniform wall thickness than IAs located elsewhere in the circle of Willis. The number of IAs in each sub-classification (i.e., smooth/rough aspect, presence or absence of blebs and/or lobules, locations and uniform/non-uniform wall thickness) lead to a low number of samples in some of the subgroups rendering statistical analyses underpowered. Another limitation to investigate a possible association between IA location and wall thickness is that some aneurysms are never treated by microsurgery preventing the inclusion of such domes for histological studies.

The prevalence of IAs is higher in women than in men, but the risk of IA rupture is not different between sexes (53, 54). In our study population, we did not find differences in IA mean, maximum or minimum wall thickness between men and women, but we showed that IA walls in females were more likely to be non-uniform in comparison to those of males. In the study performed by Kadasi et al. (35), it was shown that IA domes from women had a higher proportion of thin wall than IA domes from men. This disparity in IA wall uniformity might indicate a divergence in aneurysm remodeling between sexes, in which hormones and hemodynamic factors likely play a crucial role.

## Conclusion

Intracranial aneurysm walls are subject to a myriad of complex cellular and biochemical mechanisms resulting in a heterogeneous wall that may greatly vary from one patient to another. Quantitative analysis of IA wall thickness and thickness uniformity is paramount to better understand this disease. Considering the ensemble of patient and aneurysm characteristics used in clinical scores, perhaps the most significant finding of our study is that higher values for PHASES or ELAPSS scores were associated with higher IA wall heterogeneity. Further improvement of advanced clinical imaging techniques allowing for detailed measurement of variations in IA wall thickness may greatly help in the decision to treat or not unruptured IAs.

## Data Availability Statement

The original contributions presented in the study are included in the article/Supplementary Material, further inquiries can be directed to the corresponding author.

## Ethics Statement

The studies involving human participants were reviewed and approved by Ethical Committee of the Geneva University Hospitals and Swissethics. The patients/participants provided their written informed consent to participate in this study.

## Author Contributions

JA: acquisition of data, analysis of data, interpretation of analysis, and drafting of manuscript. AC: acquisition of data, analysis of data, interpretation of analysis, and revision of manuscript. ND, GP, BF, and JH: acquisition of data and revision of manuscript. EA: interpretation of analysis and revision of manuscript. PB: conceptualization of the study, acquisition of data, interpretation of analysis, and revision of manuscript. BK: conceptualization of the study, interpretation of analysis, and revision of manuscript. SM: design and conceptualization of the study, acquisition of data, analysis of data, interpretation of analysis, and drafting and revision of manuscript. All authors contributed to the article and approved the submitted version.

## Funding

This study was supported by grants from the Fondation Privée des HUG [to PB, EA, and BK], the Swiss SystemsX.ch initiative, evaluated by the Swiss National Science Foundation [to PB and BK], and the Swiss Heart Foundation [to BK, PB, and EA].

## Conflict of Interest

The authors declare that the research was conducted in the absence of any commercial or financial relationships that could be construed as a potential conflict of interest.

## Publisher's Note

All claims expressed in this article are solely those of the authors and do not necessarily represent those of their affiliated organizations, or those of the publisher, the editors and the reviewers. Any product that may be evaluated in this article, or claim that may be made by its manufacturer, is not guaranteed or endorsed by the publisher.
